# Use of Non-Invasive Biomarkers and Clinical Scores to Predict the Complications of Liver Cirrhosis: A Bicentric Experience

**DOI:** 10.3390/medicina60111854

**Published:** 2024-11-12

**Authors:** Giuseppe Guido Maria Scarlata, Abdulrahman Ismaiel, Maria Luisa Gambardella, Daniel Corneliu Leucuta, Francesco Luzza, Dan Lucian Dumitrascu, Ludovico Abenavoli

**Affiliations:** 1 Department of Health Sciences, University of Catanzaro “Magna Graecia”, 88100 Catanzaro, Italy; giuseppeguidomaria.scarlata@unicz.it (G.G.M.S.); marialuisa.gambardella@studenti.unicz.it (M.L.G.); luzza@unicz.it (F.L.); 2 2nd Department of Internal Medicine, “Iuliu Hatieganu” University of Medicine and Pharmacy, 400006 Cluj-Napoca, Romania; ddumitrascu@umfcluj.ro; 3 Department of Medical Informatics and Biostatistics, “Iuliu Hatieganu” University of Medicine and Pharmacy, 400012 Cluj-Napoca, Romania; dleucuta@umfcluj.ro

**Keywords:** diagnosis, management, guidelines, clinical data

## Abstract

*Background and objectives:* Liver cirrhosis is a chronic, progressive condition characterized by fibrosis and architectural distortion of the liver, leading to impaired liver function and severe complications. Accurately predicting these complications is crucial to the improvement of patient outcomes. Therefore, this study aimed to evaluate the accuracy of various non-invasive biomarkers and clinical scores in assessing the risk of complications among cirrhotic patients. *Materials and methods:* We conducted an observational retrospective study involving 236 cirrhotic patients from two tertiary care hospitals in Italy and Romania, in a timespan ranging from January 2021 to March 2024. Data on clinical characteristics, liver function tests, hematological indices, various non-invasive biomarkers, and clinical scores were collected and analyzed. Receiver operating characteristic analysis was performed to assess the accuracy of these biomarkers and clinical scores in predicting complications, including the presence of varices and hepato-renal syndrome. *Results:* The Child–Pugh score showed the highest accuracy for cirrhosis-related complications, with an area under curve (AUC) = 0.667. The red cell distribution width coefficient of variation followed closely with an AUC = 0.646. While the Child–Pugh score had a high specificity (85.42%), its sensitivity was low (37.97%). In patients with varices, non-invasive scores such as platelet distribution width (PDW) and the RDW-to-platelet ratio (RPR) showed modest predictive ability, with an AUC = 0.594. For hepato-renal syndrome, the Model for End-Stage Liver Disease (MELD) score showed the highest diagnostic accuracy with an AUC = 0.758. *Conclusions:* The most reliable biomarkers for detecting complications, varices, and hepato-renal syndrome, are, respectively, the Child–Pugh Score, PDW along with RPR, and the MELD score. However, while these scores remain valuable, the moderate diagnostic accuracy of other indices suggests the need for a more integrated approach to risk stratification. Future research should focus on validating these tools across different populations and incorporating emerging biomarkers to enhance predictive accuracy and inform more effective clinical decision-making.

## 1. Introduction

Liver cirrhosis is a chronic and progressive condition characterized by extensive scarring (fibrosis) and architectural distortion of the liver tissue resulting from sustained liver injury. This process leads to the replacement of healthy liver parenchyma with fibrotic tissue, which subsequently impairs the liver’s ability to perform its vital functions [1]. Globally, liver cirrhosis represents a significant public health challenge, being one of the leading causes of morbidity and mortality. The World Health Organization estimates that over 1 million deaths per year are attributable to cirrhosis, ranking it among the top 20 causes of death worldwide [2].

The burden of liver cirrhosis is particularly high in regions with prevalent risk factors such as chronic hepatitis B (HBV) and C (HCV) infection, alcohol abuse, and fatty liver disease [3]. Its pathogenesis is a complex interplay of chronic liver injury, inflammation, and progressive fibrosis. The liver’s response to sustained injury—whether due to viral hepatitis, alcohol, metabolic disorders, or autoimmune conditions—leads to the activation of hepatic stellate cells, which deposit extracellular matrix components that result in fibrosis. Over time, this fibrotic process disrupts normal liver architecture, leading to impaired liver function [4,5]. Although the biochemical pathways involved have not yet been fully elucidated, the gut microbiota also plays a crucial role [6,7].

Clinically, patients with cirrhosis may be asymptomatic in early stages and then display a range of symptoms, including severe manifestations [8]. Its management involves addressing the underlying cause, slowing disease progression, and managing symptoms and complications through pharmacological treatments, lifestyle modifications, and in advanced cases, liver transplantation [6,9].

Complications of liver cirrhosis are severe and often life-threatening, significantly contributing to its high mortality rate. These include portal hypertension, which can lead to esophageal varices and gastrointestinal bleeding; ascites, which increases the risk of spontaneous bacterial peritonitis; and hepatic encephalopathy, caused by the buildup of toxins in the brain. Additionally, cirrhotic patients are at increased risk of developing renal dysfunction, for instance due to hepato-renal syndrome, and coagulation disorders due to impaired synthesis of clotting factors [10].

The identification and use of biomarkers in liver cirrhosis have gained significant interest in recent years as they offer a non-invasive means of assessing liver function, staging fibrosis, and predicting complications. Biomarkers such as liver enzymes, serum albumin, bilirubin, and platelet count are commonly used in clinical practice [11]. At the same time, composite scores, like the Model for End-Stage Liver Disease (MELD) and the Child–Pugh score, incorporate multiple biomarkers and clinical parameters to assess disease severity and prognosis [12].

Despite the advancements in the understanding and use of biomarkers in liver cirrhosis, there is an urgent need to identify new potential biomarkers that can more accurately predict the onset and progression of complications. Early detection of these complications is critical in improving patient outcomes and reducing the associated morbidity and mortality. Therefore, the aim of the present study was to evaluate the use of non-invasive biomarkers and clinical scores in predicting the complications of liver cirrhosis.

## 2. Materials and Methods

### 2.1. Study Design

**Figure 1:**
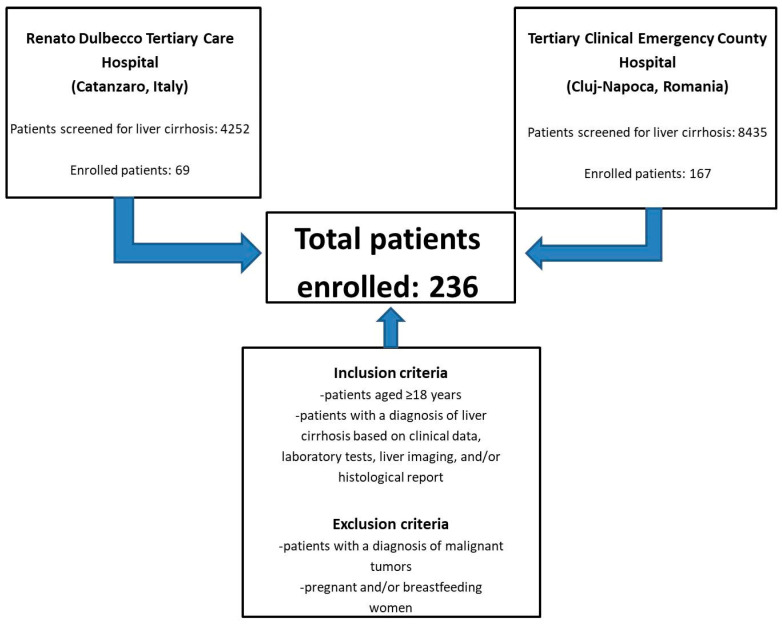
Study design workflow.

We performed an observational retrospective study, enrolling a total of 236 patients from the “Renato Dulbecco” Tertiary Care Hospital (Catanzaro, Italy) and the Tertiary Clinical Emergency County Hospital (Cluj-Napoca, Romania) over a period that spanned from 1 January 2021 to 31 March 2024. The following inclusion criteria were applied: (i) patients aged ≥18 years, (ii) patients with a diagnosis of liver cirrhosis based on clinical data, laboratory tests, liver imaging, and/or histological report [13]. The exclusion criteria were: (i) patients with a diagnosis of malignant tumors, (ii) pregnant and/or breastfeeding women (Figure 1).

### 2.2. Data Collection

Through consultation of medical records, the following data were collected: age, gender, etiology of liver cirrhosis, number of complications, presence of esophageal and gastric varices according to Baveno VII criteria [14], classification of esophageal varices according to the Japanese Research Society for Portal Hypertension guidelines [15], presence of hepato-renal syndrome according to European Association for the Study of the Liver guidelines [16], portal hypertensive gastropathy [14], portal vein ectasia and thrombosis [14], splenomegaly [14], spontaneous bacterial peritonitis [16], and previous coronavirus disease (COVID-19) infection diagnosed by real-time polymerase chain reaction. The following laboratory parameters were also evaluated: albumin, alkaline phosphatase (ALP), aspartate aminotransferase (AST), alanine aminotransferase (ALT), activated partial thromboplastin time (aPTT), creatinine, fibrinogen, γ-glutamyl transferase (GGT), international normalized ratio (INR), prothrombin time (PT), platelets, potassium, sodium, total bilirubin, red blood cell distribution width-variation coefficient (RDW-CV), and platelet distribution width (PDW). Concurrently, the following non-invasive scores were calculated: Child–Pugh, MELD, MELD Na [12], RDW-to-platelet ratio (RPR) [17], cirrhosis probability in hepatitis C (Lok) index [18], age, serum bilirubin, INR, serum creatinine (ABIC) [19], AST/ALT ratio [20], non-alcoholic fatty liver disease fibrosis (NFS) score [21], fibrosis-4-index (FIB-4) [21], aggregate systemic inflammation index (AISI) [22], neutrophil lymphocyte ratio (NLR) [23], neutrophil lymphocyte ratio to albumin ratio (NLRAR) [24], AST to platelet ratio index (APRI) [25], prognostic nutritional index (PNI) [26], King score [27], platelet lymphocyte ratio (PLR) [28], platelet-albumin-bilirubin (PALBI) [29], derived neutrophil-to-lymphocyte ratio (dNLR) [30], systemic immune-inflammation index (SII) [31], triglyceride glucose (TyG) index [32], albumin-bilirubin (ALBI) score [33], platelet-to-neutrophil ratio (PNR) [34], and lymphocyte-monocyte ratio (LMR) [35].

### 2.3. Statistical Analysis

The data were reported as medians with interquartile ranges (IQR) or as numbers with percentages (*n*, %). Clinical characteristics of the study population were compared across categorized groups using various statistical tests depending on the type of data. For normally distributed quantitative variables, an independent samples *t*-test was used, while the Wilcoxon rank-sum test was applied for non-normally distributed quantitative data. Categorical data were analyzed using chi square (χ^2^) and Fisher’s exact tests. To assess the accuracy of biomarkers and non-invasive scores in predicting complications of liver cirrhosis, receiver operating characteristic (ROC) analysis was conducted. Several logistic regression models were fitted to predict different complications for the most promising biomarkers found through ROC analysis. Two models were built for each biomarker, one with the biomarker as a continuous variable, and another with the biomarker as a binary variable (≥the cutoff value found by ROC analysis). All models were adjusted for known confounders: age, gender, HBV, HCV, and alcoholic etiology. For all models, the Hosmer and Lemeshow test was used to check the goodness of fit, while the variance inflation factor was used to check for multicollinearity. The adjusted odds ratio, the 95% confidence interval and the *p*-value were reported for each predictor of interest, as well as the area under the ROC curve of the multiple logistic regression model. A *p*-value of <0.05 was considered statistically significant. The analysis was carried out using R software version 4.1.2 (R Foundation for Statistical Computing).

## 3. Results

To evaluate whether non-invasive biomarkers and clinical scores can predict the risk of complications in 236 Italian (69/236) and Romanian (167/236) patients with liver cirrhosis, ROC analysis was performed, as shown in Table 1. The Child–Pugh score demonstrated the highest accuracy among the variables with an area under curve (AUC) of 0.667 (95% confidence interval [CI]: 0.588–0.74), with a sensitivity of 37.97% and specificity of 85.42%. For a cut-off of 2, the sensitivity is 80.75% and the specificity is 39.58%. RDW-CV followed closely with an AUC of 0.646 (95% CI: 0.559–0.731), sensitivity of 65.78%, and specificity of 58.33. Other variables including RPR and Lok index exhibited an AUC of 0.609 (95% CI: 0.523–0.698) and 0.600 (95% CI: 0.506–0.69), respectively, with moderate sensitivity and specificity. Scores such as the AST/ALT ratio and ABIC presented an AUC of 0.577 (95% CI: 0.495–0.658) and 0.579 (95% CI: 0.489–0.671), respectively. Notably, the ABIC had a high specificity of 89.58% but a low sensitivity of 12.3%. Several other parameters, including MELD Na, FIB-4, and AISI, had AUC values between 0.553 and 0.566, indicating moderate to low diagnostic accuracy. Sensitivity for these indices ranged from 23.53% to 74.33%, with specificity between 47.92% and 79.17%. Lower AUC values were observed for scores such as the NLR, APRI, PNI, and King score, with AUCs ranging from 0.531 to 0.548. Sensitivity and specificity varied among these variables. Lastly, dNLR, SII, TyG, ALBI, and LMR exhibited AUCs near 0.5.

The same analysis was performed in our cohort of cirrhotic patients to assess their ability to predict the risk of developing varices, as shown in Table 2. Specifically, PDW and RPR both exhibited an AUC of 0.594, with PDW having a sensitivity of 64.81% and specificity of 51.97%, and RPR showing a sensitivity of 26.85% and specificity of 87.4%, respectively. The AISI had a slightly lower AUC of 0.590 (95% CI: 0.516–0.662), characterized by a sensitivity of 100% but very low specificity at 1.57%. Similarly, the SII showed an AUC of 0.583, but with extremely low sensitivity of 2.78% and very high specificity of 99.21% (95% CI: 0.51–0.659). At the same time, the ABIC had an AUC of 0.580, with a low sensitivity of 13.89% and a high specificity of 88.19% (95% CI: 0.501–0.651). Among the other indices, FIB-4, Lok index, NFS, and APRI exhibited AUCs ranging from 0.560 to 0.566. These indices varied in their sensitivity (ranging from 55.56% to 78.7%) and specificity (ranging from 35.43% to 58.27%). Several indices such as the NLR, NLRAR, RDW-CV, and LMR resulted in lower AUCs between 0.539 and 0.544, with a higher sensitivity but lower specificity. The MELD score and MELD-Na had AUCs of 0.533 and 0.531 respectively, with MELD showing a higher sensitivity (83.33%) but a lower specificity (32.28%) compared to MELD-Na. Lower AUCs were observed for PNI, ALBI, and Child–Pugh score, with AUCs close to 0.5. Finally, the TyG index had the lowest AUC of 0.467 (95% CI: 0.395–0.544), with a moderate sensitivity of 41.67% and a specificity of 62.2%, respectively.

Subsequently, the same analysis was performed in our cohort of cirrhotic patients to assess their ability to predict the risk of developing hepato-renal syndrome, as shown in Table 3. The MELD score resulted in the highest diagnostic accuracy among the evaluated parameters, with an AUC of 0.758, a high sensitivity of 95.83% and a moderate specificity of 49.76% (95% CI: 0.679–0.833). The MELD Na score followed, with an AUC of 0.701 (95% CI: 0.594–0.807), a sensitivity of 58.33% and specificity of 81.52%, respectively. At the same time, the Child–Pugh score had an AUC of 0.659 (95% CI: 0.537–0.764) and a sensitivity and specificity of 75% and 52,61%, respectively. Among hematological indices, the RDW-CV showed an AUC of 0.614 (95% CI: 0.492–0.723), with a sensitivity and specificity of 70.83% and 56.87%, respectively. Similarly, the PLR exhibited an AUC of 0.608 (95% CI: 0.491–0.725), with sensitivity and specificity of 70.83% and 50.71%, respectively. Other variables, such as the ABIC, had an AUC of 0.601 (95% CI: 0.474–0.723), showing a low sensitivity of 37.5%, but a relatively high specificity of 83.89%. The PALBI score demonstrated an AUC of 0.581 (95% CI: 0.466–0.695) with a sensitivity of 75% and a specificity of 47.39%, respectively. Lower AUCs were observed for indices such as the SII and AST/ALT ratio, with AUCs of 0.569 and 0.563, respectively. These indices had varying sensitivity and specificity, indicating limited diagnostic utility in this context. Several variables, including the Lok index, LMR, PDW, and NLR, had AUCs ranging from 0.538 to 0.554. The APRI and ALBI scores had little use as diagnostic tools, as they resulted in AUCs of 0.534 and 0.522, respectively. Variables such as the NFS, TyG index, and PNI showed even lower AUCs, ranging from 0.455 to 0.502, with generally low sensitivity and specificity.

The most promising biomarkers found using ROC analysis were included in multiple logistic regression models adjusted for known confounders (age, gender, HBV, HCV, and alcoholic etiologies), as reported in Table 4. In the models predicting complications using continuous predictors, the Child–Pugh score and Lok index remained statistically significant, while the RPR and ABIC did not reach the significance threshold. In the models predicting complications using binary predictors (≥ the cut-offs values found by ROC analysis), the Child–Pugh score, the RPR, and Lok index remained statistically significant. In the models predicting the hepato-renal syndrome using continuous predictors, the MELD score, MELD Na, and the Child–Pugh score remained statistically significant. In the models predicting the hepato-renal syndrome using binary predictors (≥ the cut-offs values found by ROC analysis), the MELD score, MELD Na, the Child–Pugh score, and RDW-CV (%) remained statistically significant. In models predicting varices using continuous predictors, no biomarker was statistically significant. In models predicting varices using binary predictors (≥ the cutoffs values found by ROC analysis), PDW and RPR were statistically significant.

Finally, we report the general characteristics among the Italian and Romanian cohorts of patients in Table 5. Specifically, the median age of the Italian cohort was 63 years (IQR 55–71), which was similar to the Romanian cohort with a median age of 63 years (IQR 58.5–70), showing no significant difference (*p* = 0.931). Similarly, the proportion of males was also comparable between the two groups (*n* = 47, 68% vs. *n* = 115, 69%; *p* = 0.91). Alcoholic etiology of liver cirrhosis was significantly more prevalent among Romanian patients compared to Italian patients (*n* = 108, 65% vs. *n* = 29, 42%; *p* = 0.001). Conversely, dysmetabolic etiology was more common in the Italian group compared to the Romanian group (*n* = 17, 25% vs. *n* = 4, 2%; *p* < 0.001). Other etiologies, including autoimmune, cryptogenic, HBV-related, HCV-related, and mixed causes, showed no statistically significant differences between the two groups. The Child–Pugh B classification was more frequent in the Italian cohort (*n* = 30, 49%) compared to the Romanian cohort (*n* = 47, 36%), but this difference did not reach statistical significance (*p* = 0.073). The median MELD score was lower in the Italian group (10.36, IQR 8.67–14.73) than in the Romanian group (12.01, IQR 8.7–16.42), though this difference was not statistically significant (*p* = 0.063). However, the MELD Na score was significantly lower in the Italian cohort than in the Romanian cohort [9.66 (IQR 5.53–15.33) vs. 11.92 (IQR 7.18–17.94); *p* = 0.01]. Regarding complications, ascites was significantly more prevalent among Italian patients than Romanian patients (*n* = 42, 61% vs. *n* = 46, 27%; *p* < 0.001). Esophageal and gastric varices were also more prevalent in the Italian cohort than in the other group of patients (*n* = 7, 10% vs. *n* = 3, 2%, *p* = 0.008). Additionally, portal vein ectasia was significantly more frequent in the Italian group (*n* = 26, 38% vs. *n* = 10, 6%, *p* < 0.001), as well as splenomegaly (*n* = 44, 64% vs. *n* = 71, 42%; *p* = 0.003). Regarding laboratory parameters, Italian patients had higher median albumin levels compared to Romanian patients [3.8 g/dL (IQR 3.5–4.1) vs. 3.2 g/dL, (IQR 2.8–3.8); *p* < 0.001]. AST levels were significantly lower in the Italian group than in the Romanian group [34 UI/L (IQR 23–52) vs. 48 UI/L (IQR 31–82); *p* < 0.001], and so were GGT levels [43 UI/L (IQR 33–95) vs. 88 UI/L (IQR 43–213.5); *p* < 0.001]. The aPTT was significantly longer in Italian patients than in Romanian patients [36 s (IQR 30–41) vs. 32.7 s (IQR 29.75–35.45); *p* = 0.01]. Additionally, sodium levels were significantly higher in the Italian group compared to the Romanian group [139 mmol/L (IQR 136–141) vs. 138 mmol/L (IQR 135–140); *p* = 0.034]. Lastly, total bilirubin levels were significantly lower in the Italian cohort than the Romanian cohort [1.1 mg/dL (IQR 0.69–1.83) vs. 1.5 mg/dL (IQR 1.02–2.83); *p* < 0.001]. The same evaluation was performed stratifying the two cohorts of patients according to the presence of complications (Table S1, Supplementary Material), varices (Table S2, Supplementary Material), and hepato-renal syndrome (Table S3, Supplementary Material).

## 4. Discussion

### 4.1. Non-Invasive Biomarkers and Clinical Scores for Predicting Liver Cirrhosis Complications

Liver cirrhosis is a pathological condition that requires careful patient management in order to prevent the various complications that can arise. For this reason, it is essential to search for new non-invasive biomarkers that may aid in the early diagnosis of these complications. Our findings provide a comprehensive analysis of the predictive capabilities of various non-invasive biomarkers and clinical scores in assessing the risk of complications among patients with liver cirrhosis in Italian and Romanian cohorts. ROC analysis revealed significant variability in the diagnostic performance of these indices, emphasizing the complexity of accurately predicting complications in cirrhotic patients. In this regard, our analysis reveals that the most reliable biomarkers for detecting complications, varices, and hepato-renal syndrome, are, respectively, the Child–Pugh Score, PDW in conjunction with RPR, and the MELD score. Specifically, the Child–Pugh score, traditionally used to assess the prognosis of patients with liver cirrhosis, demonstrated the highest accuracy in predicting complications among the evaluated parameters, with an AUC of 0.667. This score’s longstanding use in clinical practice is well-established for the stratification of patients by disease severity [36]. However, in our study, while the specificity was high (85.42%), indicating that the score effectively identified patients without complications, the sensitivity was notably low (37.97%). This low sensitivity suggests that the Child–Pugh score may miss a considerable number of patients who are at risk of developing complications, thus limiting its utility in early-stage disease management or in populations with milder disease manifestations. The modest AUC value further highlights its limitations in accurately predicting complications, especially when used alone. The low sensitivity of the Child–Pugh score is problematic, as false negatives can have serious clinical implications. The specificity is relatively high, thus helping in confirming the presence of complications. Since the sensitivity is low, its use for screening is reduced in clinical environments in which failing to identify at-risk patients may lead to serious repercussions. Tailoring the threshold of the Child–Pugh score to enhance sensitivity could be a potential solution, although this may come at the cost of reduced specificity, and thus we highlight the need for a balance based on clinical priorities. For a cut-off of 2, the sensitivity is 80.75% and the specificity is 39.58%. The RDW-CV showed an AUC = 0.646, making it the second most accurate predictor of complications in this cohort. RDW-CV, a measure of the variability in red blood cell size, is emerging as a marker of systemic inflammation and poor prognosis in various chronic conditions, including liver cirrhosis [37]. In our study, the higher sensitivity (65.78%) compared to the Child–Pugh score suggests that RDW-CV is more effective in identifying patients who are likely to develop complications. However, its specificity was moderate (58.33%), implying a higher rate of false positives. The utility of RDW-CV as a non-invasive, easily obtainable biomarker makes it a promising candidate for inclusion in multi-parametric scores or as a part of routine monitoring in cirrhotic patients. The moderate AUC value reflects its potential role as a supplementary tool rather than a standalone predictor. Its role was examined in a case-control study conducted on patients with liver cirrhosis of various etiologies, showing that RDW increased with worsening Child–Pugh score and was associated with poorer hospital outcomes (AUC = 0.76) [38]. Our study also evaluated a range of additional non-invasive scores and biomarkers, including the RPR, AST/ALT ratio and ABIC, with AUCs ranging from 0.577 to 0.609. These scores, while exhibiting some diagnostic value, were generally less accurate than the Child–Pugh score and RDW-CV. The RPR combines hematological parameters, making them useful in certain contexts but insufficient as primary tools for risk prediction. However, it showed good accuracy in predicting the onset of liver fibrosis and cirrhosis in patients with chronic HBV (AUC = 0.825 and =0.884, respectively) and autoimmune hepatitis (AUC = 0.780 for significant fibrosis, AUC = 0.639 for advanced fibrosis, and AUC = 0.724 for cirrhosis) [39,40]. At the same time, the ABIC, while showing high specificity (89.58%), had an extremely low sensitivity (12.3%), highlighting its limitedness as a screening tool. Indeed, in clinical practice, its use makes it possible to stratify the risk of death in patients with alcoholic hepatitis at two different time-points (90 days and 1 year) with an AUC = 0.82 [19]. The AST/ALT ratio, often used in assessing liver inflammation and fibrosis, showed limited predictive value in our analysis, with an AUC = 0.577. This biomarker can predict liver cirrhosis in patients with chronic HCV infection and it is an independent risk factor for adverse 90-day outcomes in patients with cirrhosis and HBV-associated advanced fibrosis [41,42].

### 4.2. Non-Invasive Biomarkers and Clinical Scores for Predicting Varices and Hepato-Renal Syndrome

When analyzing the predictive power of these scores in patients with esophageal and gastric varices, the overall accuracy of the indices decreased. PDW and RPR, both with AUCs = 0.594, proved to be of modest predictive ability in this subgroup. PDW had a higher sensitivity (64.81%) but a lower specificity (51.97%), while RPR had a higher specificity (87.4%) but a lower sensitivity (26.85%). These findings suggest that in the context of varices, these markers may help in ruling out patients without complications but are less reliable in predicting who will develop such complications. However, platelet count can predict the grade of esophageal varices in cirrhotic patients, showing a negative correlation between these two variables [43]. At the same time, in association with the use of transient elastography, platelet count showed a good sensitivity and specificity in the early detection of the presence of esophageal varices in patients with infective liver cirrhosis [44]. In our study, the AISI exhibited a perfect sensitivity (100%) but a very low specificity (1.57%), while the SII had a very high specificity (99.21%) but a very poor sensitivity (2.78%). These evaluations indicate that, while these indices may be valuable in specific diagnostic contexts, they are not reliable as general predictive tools for complications in cirrhotic patients with varices [45]. Similarly, a recent study demonstrated that increased SII levels were correlated with liver steatosis but not with the subsequent conditions leading to cirrhosis [46]. A single-center study highlighted the modest accuracy of ALBI and PALBI in predicting the onset of esophageal varices in cirrhotic patients (AUC = 0.603 and =0.606, respectively) [47]. In contrast, our study demonstrated even lower accuracy for these biomarkers (AUC between 0.4 and 0.5). At the same time, another study showed an accuracy between 0.5 and 0.6 for APRI, NFS, FIB-4, and King score, according to our results [48]. Regarding the hepato-renal syndrome, the predictive performance of the MELD score was the highest of the evaluated indices, with an AUC = 0.758. The MELD score’s high sensitivity (95.83%) underlines its effectiveness in identifying patients at high risk for this severe complication, although its moderate specificity (49.76%) suggests a high rate of false positives. This makes the MELD score particularly useful in a clinical setting where the early detection of hepato-renal syndrome is crucial for timely intervention [49]. Indeed, patients with this complication and higher MELD levels had the worst outcome [50]. The MELD Na score showed an AUC = 0.701 and higher specificity (81.52%) compared to the MELD score alone. This finding aligns with previous studies that have identified serum sodium as a critical factor in the prognosis of cirrhotic patients [51]. The improved specificity of the MELD Na score suggests that it may be more effective in accurately predicting patients who are at a risk of hepato-renal syndrome, reducing the potential for unnecessary interventions. The Child–Pugh score, with an AUC = 0.659 in this subgroup, showed balanced sensitivity (75%) and specificity (52.61%). While this indicates that it remains a useful tool, it is less effective than the MELD and MELD Na scores in predicting hepato-renal syndrome [12]. Among hematological indices, RDW-CV and PLR showed moderate predictive value (AUCs = 0.614 and =0.608, respectively), indicating their potential usefulness as supplementary biomarkers in this specific clinical context. This finding is in agreement with a recent study showing that hematological indices including PLR can moderately predict the occurrence of complications [52].

### 4.3. Comparison Between Italian and Romanian Cohorts

The significant differences observed between the Italian and Romanian patient cohorts provide valuable insights into how cirrhosis etiology and management may influence disease progression and complication risk. The Romanian cohort had a significantly higher prevalence of alcohol-related liver cirrhosis, which may contribute to a more aggressive disease progression and a higher burden of complications, typically associated with alcohol-related liver disease [53,54]. Conversely, the Italian cohort had a higher prevalence of dysmetabolic liver cirrhosis, which might be linked to lifestyle and metabolic factors prevalent in this population [55]. The Italian patients also presented more advanced disease features, such as higher rates of ascites, varices, and portal vein ectasia, despite having better-preserved liver function, as indicated by the higher albumin levels and lower AST and GGT levels. These findings highlight the differences in the underlying etiology of liver cirrhosis which could lead to variations in disease presentation [56]. The significant differences in laboratory parameters, such as the higher sodium levels and lower total bilirubin levels in the Italian group, could reflect differences in clinical practices or the stage of disease at which patients are diagnosed and treated (Table 6) [57]. These differences underscore the importance of considering demographic and regional factors when assessing liver cirrhosis patients and tailoring management strategies accordingly.

### 4.4. Limitations and Implications for Future Research

Despite the high number of biomarkers evaluated and the insights provided by this study, several limitations must be acknowledged. The modest AUC values for many of the indices suggest that they have limited usefulness as standalone predictors of complications in liver cirrhosis patients. Furthermore, the study’s modest sample was confined to Italian and Romanian populations, potentially limiting the applicability of these findings to other ethnic or geographic groups. Moreover, we have not applied other diagnostic algorithms which include imaging techniques and the products of extracellular matrix (ECM) metabolism. In this regard, recent literature highlighted the integration of specific biomarkers, such as hyaluronic acid, procollagen III, and matrix metalloproteinases, along with imaging assays like magnetic resonance imaging (MRI), to differentiate between early-stage and mild fibrosis [58]. These biomarkers reflect various aspects of ECM turnover and tissue remodeling, which are crucial in the progression of fibrosis. Recent studies have employed algorithms that analyze the products of ECM metabolism, including collagen degradation and deposition rates, to improve diagnostic precision [59]. For instance, the Enhanced Liver Fibrosis score, which combines multiple serum biomarkers, has been validated for detecting early fibrosis stages [60]. By integrating these biomarkers with advanced imaging techniques, researchers aim to improve both sensitivity and specificity in fibrosis detection, allowing for more accurate disease staging and timely therapeutic interventions. Future research should focus on larger, more diverse populations to confirm these findings and explore the potential of combining multiple biomarkers and clinical scores to enhance predictive accuracy [61,62]. Additionally, integrating novel biomarkers from fields such as genomics, proteomics, and metabolomics could provide deeper insights into the pathophysiology of liver cirrhosis and improve the ability to predict complications [7]. Advanced imaging techniques and machine learning algorithms may also offer new avenues for improving the accuracy and utility of non-invasive predictive tools [63]. Incorporating the predictive capabilities of non-invasive biomarkers and clinical scores into routine clinical workflows is of crucial importance for the improvement of the management of liver cirrhosis complications. Given the varying diagnostic accuracy of these tools, we propose a tiered approach where the Child–Pugh Score, PDW in conjunction with RPR, and the MELD score can be employed at different stages of disease progression. These scores can help guide clinical decision-making, particularly in identifying risks for varices and hepato-renal syndrome. To optimize their utility, clinicians should conduct periodic assessments and adjust management plans as these scores evolve over time, ensuring a more personalized approach to patient care. Additionally, combining these established scores with emerging biomarkers may further enhance predictive accuracy and facilitate the development of composite models. Such integrated risk stratification approaches will better capture the complexity of cirrhosis progression, ultimately improving patient outcomes.

## 5. Conclusions

Our analysis shows that although new biomarkers are being studied in the context of liver cirrhosis, old ones remain essential. This study highlights the varying predictive capabilities of non-invasive biomarkers and clinical scores for complications in liver cirrhosis, showing how the most reliable biomarkers for detecting complications, varices, and hepato-renal syndrome, are, respectively, the Child–Pugh Score, PDW in conjunction with RPR, and the MELD score. However, while these scores remain valuable, the moderate diagnostic accuracy of other indices suggests the need for a more integrated approach to risk stratification. Understanding the differences in patient populations and in the etiology of cirrhosis is essential in improving the clinical and therapeutic management of this complex condition. Furthermore, the study underscores the importance of addressing the limitations in sensitivity and specificity of current scoring systems. Relying solely on individual scores may overlook critical variations in disease progression, which necessitate the development of composite models or personalized risk assessments. Future research should focus on validating these tools across different populations and on incorporating emerging biomarkers to enhance predictive accuracy and to inform more effective clinical decision-making.

## Figures and Tables

**Table 1:** ROC analysis classifying the presence of complications in 236 patients with liver cirrhosis.

Variable	AUC (95% CI)	*p*-Value	Sensitivity	Specificity	Cut-Off
Child–Pugh score	0.667 (0.588–0.74)	<0.001	37.97	85.42	8
RDW-CV (%)	0.646 (0.559–0.731)	0.001	65.78	58.33	14
RPR	0.609 (0.523–0.698)	0.015	69.52	54.17	0.09
Lok index	0.6 (0.506–0.69)	0.033	62.03	64.58	0.836922
ABIC	0.579 (0.489–0.671)	0.089	12.3	89.58	9.2246
AST/ALT ratio	0.577 (0.495–0.658)	0.064	31.55	91.67	2.153846
NFS	0.566 (0.465–0.658)	0.18	65.24	52.08	2.91
FIB-4	0.562 (0.472–0.647)	0.165	50.8	62.5	5.124717
MELD Na	0.561 (0.466–0.654)	0.203	74.33	47.92	7.201916
AISI	0.553 (0.462–0.641)	0.246	23.53	79.17	473.6545
NLRAR	0.548 (0.452–0.643)	0.325	75.94	37.5	0.53
NLR	0.542 (0.452–0.631)	0.358	80.75	33.33	1.73
APRI	0.54 (0.455–0.63)	0.37	16.58	95.83	2.984127
PDW (fL)	0.536 (0.451–0.618)	0.398	48.66	64.58	16.2
PNI	0.536 (0.436–0.635)	0.478	86.1	20.83	27.24
King score	0.534 (0.442–0.629)	0.476	62.03	52.08	24.48573
PLR	0.531 (0.44–0.623)	0.507	40.11	72.92	134.5
PALBI	0.523 (0.433–0.617)	0.624	45.45	66.67	−3.82772
MELD score	0.52 (0.419–0.621)	0.698	67.91	47.92	9.627471
dNLR	0.519 (0.429–0.61)	0.681	90.37	16.67	0.96
SII	0.515 (0.422–0.606)	0.749	36.36	70.83	591.81
TyG	0.513 (0.416–0.614)	0.797	99.47	6.25	3.30298
ALBI	0.512 (0.411–0.611)	0.814	85.56	27.08	−1.77091
PNR	0.504 (0.403–0.596)	0.935	57.75	52.08	29.92
LMR	0.497 (0.409–0.589)	0.948	33.16	75	3.56

Abbreviations: RDW-CV, red blood cell distribution width-variation coefficient, RPR, RDW-to-platelet ratio, RDW-to-platelet ratio; PDW, platelet distribution width; MELD, Model for End-Stage Liver Disease; Lok, cirrhosis probability in hepatitis C; ABIC, age, serum bilirubin, INR, and serum creatinine; AST/ALT, aspartate aminotransferase/alanine aminotransferase; NFS, non-alcoholic fatty liver disease fibrosis; FIB-4, fibrosis index, fibrosis-1-index; NLR, neutrophil lymphocyte ratio; NLRAR, neutrophil lymphocyte ratio to albumin ratio; PLR, platelet lymphocyte ratio; APRI, AST to platelet ratio index; PALBI, platelet-albumin-bilirubin; dNLR, derived neutrophil-to-lymphocyte ratio; SII, systemic immune-inflammation index; PNI, prognostic nutritional index; TyG, triglyceride glucose index; ALBI, albumin-bilirubin; PNR, platelet-to-neutrophil ratio; LMR, lymphocyte-monocyte ratio; AUC, area under curve; CI, confidence interval.

**Table 2:** ROC analysis classifying the presence of varices in 236 patients with liver cirrhosis.

Variable	AUC (95% CI)	*p*-Value	Sensitivity	Specificity	Cut-Off
PDW (fL)	0.594 (0.52–0.668)	0.013	64.81	51.97	16.1
RPR	0.594 (0.52–0.663)	0.01	26.85	87.4	0.21
AISI	0.59 (0.516–0.662)	0.016	100	1.57	5.470225
SII	0.583 (0.51–0.659)	0.029	2.78	99.21	3764.95
ABIC	0.58 (0.501–0.651)	0.037	13.89	88.19	9.2106
FIB-4	0.566 (0.495–0.636)	0.067	78.7	36.22	2.758857
dNLR	0.562 (0.49–0.638)	0.101	89.81	11.81	0.96
Lok index	0.562 (0.49–0.633)	0.089	60.19	55.91	0.890572
NFS	0.562 (0.489–0.633)	0.091	55.56	58.27	3.33
APRI	0.56 (0.486–0.633)	0.11	77.78	35.43	0.634072
PLR	0.552 (0.486–0.624)	0.14	94.44	7.09	33.22
King score	0.549 (0.478–0.621)	0.179	65.74	51.18	26.39189
NLR	0.544 (0.468–0.62)	0.256	87.04	16.54	1.46
NLRAR	0.542 (0.47–0.615)	0.256	75	28.35	0.53
RDW-CV (%)	0.541 (0.465–0.614)	0.281	39.81	71.65	15.1
LMR	0.539 (0.468–0.613)	0.292	59.26	52.76	2.66
AST/ALT ratio	0.538 (0.465–0.611)	0.308	36.11	77.17	2.090909
MELD score	0.533 (0.459–0.606)	0.379	83.33	32.28	8.653332
MELD Na	0.531 (0.456–0.602)	0.405	61.11	51.18	10.25069
PNR	0.53 (0.461–0.604)	0.411	90.74	17.32	16.54
PALBI	0.512 (0.438–0.586)	0.751	89.81	17.32	−4.07507
PNI	0.497 (0.421–0.574)	0.939	84.26	23.62	28.64
ALBI	0.496 (0.418–0.571)	0.918	89.81	23.62	−1.74042
Child–Pugh score	0.494 (0.417–0.571)	0.879	24.07	82.68	9
TyG	0.467 (0.395–0.544)	0.385	41.67	62.2	3.740797

Abbreviations: RDW-CV, red blood cell distribution width-variation coefficient, RPR, RDW-to-platelet ratio; PDW, platelet distribution width; MELD, Model for End-Stage Liver Disease; Lok, cirrhosis probability in hepatitis C; ABIC, age, serum bilirubin, INR, and serum creatinine; AST/ALT, aspartate aminotransferase/alanine aminotransferase; NFS, non-alcoholic fatty liver disease fibrosis; FIB-4, fibrosis index, fibrosis-1-index; NLR, neutrophil lymphocyte ratio; NLRAR, neutrophil lymphocyte ratio to albumin ratio; PLR, platelet lymphocyte ratio; APRI, AST to platelet ratio index; PALBI, platelet-albumin-bilirubin; dNLR, derived neutrophil-to-lymphocyte ratio; SII, systemic immune-inflammation index; PNI, prognostic nutritional index; TyG, triglyceride glucose index; ALBI, albumin-bilirubin; PNR, platelet-to-neutrophil ratio; LMR, lymphocyte-monocyte ratio; AUC, area under curve; CI, confidence interval.

**Table 3:** ROC analysis classifying the presence of hepato-renal syndrome in 236 patients with liver cirrhosis.

Variable	AUC (95% CI)	*p*-Value	Sensitivity	Specificity	Cut-Off
MELD score	0.758 (0.679–0.833)	<0.001	95.83	49.76	10.66849
MELD Na	0.701 (0.594–0.807)	<0.001	58.33	81.52	17.4982
Child–Pugh score	0.659 (0.537–0.764)	0.006	75	52.61	7
RDW-CV (%)	0.614 (0.492–0.723)	0.053	70.83	56.87	14.4
PLR	0.608 (0.491–0.725)	0.07	70.83	50.71	97.67
ABIC	0.601 (0.474–0.723)	0.112	37.5	83.89	8.9316
PALBI	0.581 (0.466–0.695)	0.166	75	47.39	−3.87545
SII	0.569 (0.43–0.699)	0.315	45.83	75.36	749.8
AST/ALT ratio	0.563 (0.454–0.675)	0.264	66.67	48.34	1.571429
Lok index	0.554 (0.433–0.671)	0.374	58.33	62.09	0.942399
LMR	0.549 (0.413–0.678)	0.469	25	82.94	4.9
PDW (fL)	0.548 (0.42–0.668)	0.448	20.83	85.78	16.8
NLR	0.538 (0.401–0.675)	0.587	33.33	83.89	6.95
NLRAR	0.534 (0.404–0.662)	0.605	29.17	87.68	2.36
APRI	0.534 (0.398–0.673)	0.628	16.67	92.42	3.793103
ALBI	0.522 (0.413–0.638)	0.702	66.67	45.5	−1.17265
FIB-4	0.518 (0.385–0.652)	0.792	37.5	70.62	6.580626
TyG	0.514 (0.405–0.633)	0.81	75	34.6	3.603902
PNI	0.502 (0.397–0.613)	0.971	91.67	24.17	29.02
King score	0.496 (0.369–0.616)	0.949	41.67	69.67	50.24444
dNLR	0.487 (0.355–0.625)	0.85	33.33	84.36	3.66
PNR	0.483 (0.355–0.618)	0.8	37.5	81.99	57.6
RPR	0.48 (0.347–0.612)	0.767	16.67	93.36	0.31
AISI	0.472 (0.332–0.601)	0.683	37.5	81.99	533.2959
NFS	0.455 (0.344–0.573)	0.441	41.67	67.3	3.68

Abbreviations: RDW-CV, red blood cell distribution width-variation coefficient, RPR, RDW-to-platelet ratio; PDW, platelet distribution width; MELD, Model for End-Stage Liver Disease; Lok, cirrhosis probability in hepatitis C; ABIC, age, serum bilirubin, INR, and serum creatinine; ASP/ALT, aspartate aminotransferase/alanine aminotransferase; NFS, non-alcoholic fatty liver disease fibrosis; FIB-4, fibrosis index, fibrosis-1-index; NLR, neutrophil lymphocyte ratio; NLRAR, neutrophil lymphocyte ratio to albumin ratio; PLR, platelet lymphocyte ratio; APRI, AST to platelet ratio index; PALBI, platelet-albumin-bilirubin; dNLR, derived neutrophil-to-lymphocyte ratio; SII, systemic immune-inflammation index; PNI, prognostic nutritional index; TyG, triglyceride glucose index; ALBI, albumin-bilirubin; PNR, platelet-to-neutrophil ratio; LMR, lymphocyte-monocyte ratio; AUC, area under curve; CI, confidence interval.

**Table 4:** Multiple logistic regressions models predicting complications for different characteristics, adjusted for age, gender, HBV and HCV, and alcoholic etiologies.

Characteristic	OR Adjusted *	(95% CI)	*p*-Value	Model AUC	OR Adjusted **	(95% CI)	*p*-Value	Model AUC
Predicting complications								
Child.Pugh score	1.38	(1.16–1.67)	<0.001	69.17 (61.27–77.07)	2.46	(1.26–4.98)	0.01	64.35 (55.58–73.12)
RPR	56.2	(1.49–5633.44)	0.06	63.57 (55.03–72.11)	2.6	(1.33–5.1)	0.005	65.33 (56.78–73.88)
Lok index	3.96	(1.12–13.82)	0.031	63.79 (55.41–72.17)	3.09	(1.58–6.23)	0.001	66.99 (58.61–75.37)
ABIC	0.82	(0.66–1.02)	0.073	59.73 (50.98–68.49)	0.82	(0.66–1.02)	0.073	59.73 (50.98–68.49)
Predicting hepato-renal syndrome								
MELD score	1.13	(1.07–1.21)	<0.001	76.34 (67.54–85.15)	27.44	(5.46–501.13)	0.001	75.96 (68.08–83.84)
MELD Na	1.1	(1.05–1.16)	< 0.001	72.95 (62.71–83.19)	7.04	(2.81–18.64)	<0.001	74.15 (62.69–85.61)
Child–Pugh score	1.27	(1.05–1.54)	0.013	67.23 (56.89–77.57)	4.16	(1.37–18.09)	0.025	65.04 (54.75–75.33)
RDW-CV (%)	1.04	(0.94–1.13)	0.35	60.55 (47.75–73.34)	2.94	(1.2–7.99)	0.024	65.25 (54.05–76.46)
Predicting varices								
PDW (fL)	1.06	(0.98–1.36)	0.571	61.33 (53.97–68.68)	2.03	(1.17–3.56)	0.012	64.22 (57.11–71.33)
RPR	1.9	(0.31–13.66)	0.489	59.92 (52.55–67.3)	2.38	(1.22–4.75)	0.012	2.38
AISI	1	(1–1)	0.887	59.2 (51.79–66.61)	NC	NC	NC	NC

* models using the predictor as a continuous variable; **, models using the predictor as a binary variable ≥ the cutoff found by ROC analysis. Abbreviations: OR, odds ratio; AUC, area under the curve; CI, confidence interval; NC, cannot be computed; RPR, RDW-to-platelet ratio; Lok, cirrhosis probability in hepatitis C; ABIC, age, serum bilirubin, INR, and serum creatinine; RDW-CV, PDW, red blood cell distribution width-variation coefficient; platelet distribution width; AISI, aggregate systemic inflammation index.

**Table 5:** Comparison of the general characteristics of the Italian and Romanian cohort of patients.

	Italian (*n* = 69)	Romanian (*n* = 167)	*p*-Value
Demographic data			
Age (years), median (IQR)	63 (55–71)	63 (58.5–70)	0.931
Male gender, *n* (%)	47 (68)	115 (69)	0.91
Clinical data			
Alcoholic, *n* (%)	29 (42)	108 (65)	0.001
Autoimmune, *n* (%)	3 (4)	10 (6)	0.761
Cryptogenic, *n* (%)	6 (9)	7 (4)	0.209
Dysmetabolic, *n* (%)	17 (25)	4 (2)	<0.001
HBV-related, *n* (%)	5 (7)	16 (9)	0.567
HCV-related, *n* (%)	9 (13)	33 (20)	0.22
Hemochromatosis, *n* (%)	1 (1)	3 (2)	1.00
Mixed, *n* (%)	2 (3)	13 (8)	0.242
Child–Pugh B, *n* (%)	30 (49)	47 (36)	0.073
MELD score, median (IQR)	10.36 (8.67–14.73)	12.01 (8.7–16.42)	0.063
MELD Na, median (IQR)	9.66 (5.53–15.33)	11.92 (7.18–17.94)	0.01
Previous COVID-19, *n* (%)	0	6 (3)	0.184
Complications, *n* (%)	59 (85)	129 (77)	0.152
Ascites, *n* (%)	42 (61)	46 (27)	<0.001
Presence of varices, *n* (%)	31 (45)	78 (47)	0.803
Esophageal and gastric varices, *n* (%)	7 (10)	3 (2)	0.008
Esophageal varices F1, *n* (%)	20 (29)	47 (28)	0.896
Esophageal varices F2, *n* (%)	8 (12)	33 (20)	0.132
Esophageal varices F3, *n* (%)	5 (7)	7 (4)	0.34
Gastric varices, *n* (%)	7 (10)	4 (2)	0.016
Hepato-renal syndrome, *n* (%)	11 (16)	13 (8)	0.059
Portal hypertensive gastropathy, *n* (%)	31 (45)	58 (35)	0.142
Portal vein ectasia, *n* (%)	26 (38)	10 (6)	<0.001
Portal vein thrombosis, *n* (%)	2 (3)	14 (8)	0.161
Splenomegaly, *n* (%)	44 (64)	71 (42)	0.003
Spontaneous bacterial peritonitis, *n* (%)	0	1 (0.6)	1.00
Laboratory parameters, median (IQR)			
Albumin (g/dL)	3.8 (3.5–4.1)	3.2 (2.8–3.8)	<0.001
ALP (UI/L)	111 (71–142)	101 (76–144.5)	0.913
AST (UI/L)	34 (23–52)	48 (31–82)	<0.001
ALT (UI/L)	25 (18–38)	28 (18–44)	0.254
GGT (UI/L)	43 (33–95)	88 (43–213.5)	<0.001
Platelets (10^3^/μL)	105 (78–150)	137 (86–203)	0.06
PT (s)	14 (12–16)	14.3 (12.1–17.45)	0.46
aPTT (s)	36 (30–41)	32.7 (29.75–35.45)	0.01
INR	1.24 (1.1–1.5)	1.28 (1.08–1.56)	0.467
Fibrinogen (mg/dL)	256 (205–310)	243 (197.5–316)	0.716
Creatinine (mg/dL)	0.82 (0.68–1.05)	0.79 (0.64–1.08)	0.465
Potassium (mmol/L)	4.1 (3.8–4.5)	4.17 (3.74–4.5)	0.994
Sodium (mmol/L)	139 (136–141)	138 (135–140)	0.034
Total bilirubin. (mg/dL)	1.1 (0.69–1.83)	1.5 (1.02–2.83)	<0.001

Abbreviations: HBV, Hepatitis B virus; HCV, Hepatitis C virus; MELD, Model for End-Stage Liver Disease; Coronavirus Disease 2019, COVID-19; IQR, interquartile range; ALP, alkaline phosphatase; AST, aspartate aminotransferase; ALT, alanine aminotransferase; GGT, γ-glutamyl transferase; PT, prothrombin time; aPTT, activated partial thromboplastin time; INR, international normalized ratio.

**Table 6:** Summary of the different clinical highlights in the Italian and Romanian cohorts of patients.

	Italian Cohort	Romanian Cohort
Liver cirrhosis etiology	Higher prevalence of dysmetabolic liver cirrhosis	Higher prevalence of alcohol-related liver cirrhosis
Disease progression	More advanced disease features despite better-preserved liver function	More aggressive disease progression due to alcohol-related cirrhosis
Complications	Higher rates of ascites, varices, and portal vein ectasia	Higher burden of complications
Liver function	Better preserved (higher albumin levels, lower AST and GGT levels)	Worse liver function (lower albumin levels, higher AST and GGT levels)

## Data Availability

The original contributions presented in the study are included in the article; further inquiries can be directed to the corresponding authors.

## Supplementary Materials

The following supporting information can be downloaded at: https://www.mdpi.com/article/10.3390/medicina60111854/s1, Table S1: Comparisons of participants’ characteristics based on the presence of liver cirrhosis’ complications; Table S2:. Comparisons of participants’ characteristics based on the presence of varices; Table S3: Comparisons of participants’ characteristics based on the presence of hepato-renal syndrome.
