# Supplementary material for: Use of Non-Invasive Biomarkers and Clinical Scores to Predict the Complications of Liver Cirrhosis: A Bicentric Experience
Source: Medicina (Kaunas). 2024 Nov 12;60(11):1854. doi: 10.3390/medicina60111854 (PMC11596259; doi:10.3390/medicina60111854)
Supplement: Supplementary file 1 [file medicina-60-01854-s001.zip › medicina-3242154-supplementary-Table S3.pdf]

Table S3. Comparisons of participants characteristics based on the presence of hepato-renal syndrome.

	Absence of hepato-renal syndrome (*n*=211)	Presence of hepato-renal syndrome (*n*=24)	*p*-value
**Demographic data**			
Age (years), median (IQR)	63 (58-70)	62.5 (58.75-72)	0.922
Male gender, n (%)	144 (68)	17 (71)	0.796
**Clinical data, n (%)**			
Alcoholic	124 (59)	13 (54)	0.665
Autoimmune	13 (6)	0	0.372
Cryptogenic	12 (6)	1 (4)	1.00
Dysmetabolic	17 (8)	4 (17)	0.245
HBV-related	18 (8)	2 (8)	1.00
HCV-related	39 (18)	3 (12)	0.584
Hemochromatosis	3 (1)	1 (4)	0.352
Mixed	14 (7)	1 (4)	1.00
Ascites	70 (33)	18 (75)	**<0.001**
Esophageal and gastric varices	7 (3)	3 (12)	0.07
Esophageal varices F1	56 (27)	11 (46)	**0.047**
Esophageal varices F2	37 (18)	3 (12)	0.775
Esophageal varices F3	11 (5)	1 (4)	1.00
Gastric varices	8 (4)	3 (12)	0.089
Portal hypertensive gastropathy	78 (37)	10 (42)	0.652
Portal vein ectasia	29 (14)	7 (29)	0.067
Portal vein thrombosis	13 (6)	3 (12)	0.215
Splenomegaly	101 (48)	14 (58)	0.331
Hepatic encephalopathy	46 (22)	7 (29)	0.413
Spontaneous bacterial peritonitis	0	1 (4)	0.102
**Laboratory parameters and**			

**scores, median (IQR)**			
Albumin (g/dL)	3.5 (2.9-3.95)	3.45 (3.18-3.67)	0.914
ALP (UI/L)	101 (74-141.5)	121.5 (104.25-157.25)	**0.04**
AST (UI/L)	45 (28-66)	37.5 (23.75-94.75)	0.774
ALT (UI/L)	27 (18-41)	23 (15.75-38.75)	0.413
GGT (UI/L)	80 (40-150)	61.5 (32-235.75)	0.939
Platelets ($10^3/\mu L$)	120 (81.5-176)	144.5 (84.5-223.25)	0.472
PT (s)	14 (12-17)	14.15 (12.5-17.1)	0.657
aPTT (s)	32.9 (29.75-37)	34.7 (30-37.85)	0.59
INR	1.26 (1.08-1.52)	1.27 (1.12-1.55)	0.477
Fibrinogen (mg/dL)	246 (200-313)	250.2 (183.75-331.25)	0.79
Creatinine (mg/dL)	0.76 (0.64-0.94)	1.57 (1.33-1.82)	**<0.001**
Potassium (mmol/L)	4.14 (3.75-4.47)	4.28 (3.6-4.81)	0.549
Sodium (mmol/L)	138 (135-140)	136 (133.75-139.25)	0.169
Total bilirubin. (mg/dL)	1.29 (0.84-2.32)	1.57 (0.88-3.01)	0.28
Neutrophils (10^9/L)	3.63 (2.48-5.54)	3.69 (2.13-5.46)	0.73
Lymphocytes (10^9/L)	1.26 (0.75-1.83)	1.33 (0.56-1.57)	0.301
Leucocytes (10^9/L)	5.66 (4.2-8.03)	5.64 (3.98-6.8)	0.755
Monocytes (10^9/L)	0.45 (0.32-0.61)	0.43 (0.26-0.69)	0.669
Basophils (10^9/L)	0.02 (0.01-0.04)	0.02 (0.01-0.04)	0.487
Triglycerides (mg/dL)	93 (70.5-115.5)	95.5 (69.75-134.25)	0.78
Glycemia (mg/dL)	104 (92.5-126.5)	104.5 (87.75-120.25)	0.622
Child-Pugh	7 (6-9)	9 (7.75-10)	**0.01**
MELD score,	10.85 (8.47-15.16)	16.27 (12.4-18.61)	**<0.001**
MELD Na	11.1 (6.43-16.15)	17.94 (11.18-20.92)	**0.001**
RDW-CV (%)	14.3 (13.5-15.65)	15.05 (13.75-16.52)	0.067
PDW (fL)	16.2 (15.8-16.6)	16.1 (15.67-16.42)	0.439
TyG	3.68 (3.56-3.85)	3.69 (3.6-3.81)	0.827
PNI	35.39 (29.31-40.25)	35.02 (32.13-36.98)	0.97
RPR	0.12 (0.08-0.18)	0.12 (0.07-0.18)	0.755
NLR	3.04 (1.93-5.39)	3.8 (1.68-8.12)	0.539

dNLR	1.97 (1.39-2.93)	1.94 (1.18-3.98)	0.833
PLR	97.5 (61.72-162.73)	135.82 (75.31-222.77)	0.083
LMR	2.81 (1.77-4.22)	2.24 (1.66-3.94)	0.433
PNR	32.81 (22.34-50.08)	30.16 (20.81-58.24)	0.788
SII	387.56 (171.16-737.82)	607.32 (197.29-1120.6)	0.272
AISI	151.21 (56.88-431.66)	148.58 (55.99-766.88)	0.658
NLRAR	0.92 (0.52-1.67)	0.96 (0.54-2.5)	0.587
ALBI	-1 (-1.61 - -0.26)	-0.79 (-1.35 - -0.46)	0.72
AST/ALT ratio	1.6 (1.18-2.22)	1.66 (1.31-2.32)	0.316
APRI	1.19 (0.58-2.06)	0.76 (0.38-2.18)	0.589
PALBI	-3.86 (-3.99 - -3.73)	-3.84 (-3.89 - -3.7)	0.196
FIB-4	4.76 (2.52-7.81)	4.16 (2.23-7.71)	0.774
ABIC	7.89 (7.38-8.52)	8.16 (7.56-9.12)	0.105
NFS	3.26 (2.5-3.92)	3.26 (2.03-3.77)	0.475
King score	31.7 (15.14-65.03)	32.61 (12.84-63.66)	0.953
Lok index	0.89 (0.62-0.98)	0.95 (0.68-0.99)	0.39

Abbreviations: HBV, Hepatitis B virus; HCV, Hepatitis C virus; ALP, alkaline phosphatase; AST, aspartate aminotransferase; ALT, alanine aminotransferase; GGT, γ-glutamyl transferase; PT, prothrombin time; aPTT, activated partial thromboplastin time; INR, international normalized ratio; RDW-CV, red blood cell distribution width-variation coefficient, RPR, RDW-to-platelet ratio; PDW, platelet distribution width; MELD, Model for End-Stage Liver Disease; Lok, cirrhosis probability in hepatitis C ; ABIC, age, serum bilirubin, INR, and serum creatinine; ASP/ALT, aspartate aminotransferase/alanine aminotransferase; NFS, Non-Alcoholic Fatty Liver Disease Fibrosis; FIB-4, fibrosis index, fibrosis-1-index; ASI, aggregate systemic inflammation index; NLR, neutrophil lymphocyte ratio; NLRAR, neutrophil lymphocyte ratio to albumin ratio; PLR, platelet lymphocyte ratio; APRI, AST to platelet ratio index; PALBI, platelet-albumin-bilirubin; dNLR, derived neutrophil-to-lymphocyte ratio; SII, systemic immune-inflammation index; PNI, prognostic nutritional index; TyG, triglyceride glucose index; ALBI, albumin-bilirubin; PNR, platelet-to-neutrophil ratio; LMR, lymphocyte-monocyte ratio.
