# Supplementary material for: Use of Non-Invasive Biomarkers and Clinical Scores to Predict the Complications of Liver Cirrhosis: A Bicentric Experience
Source: Medicina (Kaunas). 2024 Nov 12;60(11):1854. doi: 10.3390/medicina60111854 (PMC11596259; doi:10.3390/medicina60111854)
Supplement: Supplementary file 1 [file medicina-60-01854-s001.zip › medicina-3242154-supplementary-Table S2.pdf]

Table S2. Comparisons of participants characteristics based on the presence of varices.

	**Absence of varices** (*n*=127)	**Presence of varices** (*n*=108)	***p*-value**
**Demographic data**			
Age (years), median (IQR)	65 (59.5-70)	63 (55-68.25)	0.083
Male gender, n (%)	83 (65)	78 (72)	0.259
**Clinical data, n (%)**			
Alcoholic	78 (61)	59 (55)	0.293
Autoimmune	8 (6)	5 (5)	0.577
Cryptogenic	7 (5)	6 (6)	0.988
Dysmetabolic	10 (8)	11 (10)	0.536
HBV-related	12 (9)	8 (7)	0.576
HCV-related	20 (16)	22 (20)	0.357
Hemochromatosis	1 (1)	3 (3)	0.336
Mixed	8 (6)	7 (7)	0.955
Ascites	48 (38)	40 (37)	0.905
Esophageal and gastric varices	0	10 (9)	**<0.001**
Esophageal varices F1, n (%)	0	66 (61)	**<0.001**
Esophageal varices F2	0	40 (37)	**<0.001**
Esophageal varices F3	0	12 (11)	**<0.001**
Gastric varices, n (%)	0	9 (8)	**0.015**
Hepato-renal syndrome	9 (7)	15 (14)	0.086
Portal hypertensive gastropathy	37 (29)	51 (47)	**0.004**
Portal vein ectasia	21 (16)	15 (14)	0.575
Portal vein thrombosis	9 (7)	7 (6)	0.854
Splenomegaly	56 (44)	59 (55)	0.107
Hepatic encephalopathy	29 (23)	24 (22)	0.911
Spontaneous bacterial	1 (1)	0	1.00

peritonitis

**Laboratory parameters and scores, median (IQR)**			
Albumin (g/dL)	3.4 (2.8-4)	3.5 (2.98-3.9)	0.934
ALP (UI/L)	104 (77.5-144.5)	102.5 (71-143)	0.705
AST (UI/L)	45 (28-62.5)	42 (27-88.25)	0.796
ALT (UI/L)	26 (19-38.5)	27.5 (17-45)	0.983
GGT (UI/L)	88 (43.5-202)	57.5 (33-145.75)	**0.025**
Platelets (10^3/μL)	140 (90.5-209)	111 (77-164.75)	**0.025**
PT (s)	13.9 (12-17)	14.05 (12.28-17)	0.51
aPTT (s)	32.9 (29.9-36.5)	33.15 (29.67-38.02)	0.374
INR	1.23 (1.08-1.54)	1.28 (1.1-1.53)	0.41
Fibrinogen (mg/dL)	256 (203.5-329.5)	241.2 (193.5-302)	0.071
Creatinine (mg/dL)	0.79 (0.66-1.08)	0.79 (0.65-1.06)	0.921
Potassium (mmol/L)	4.07 (3.72-4.38)	4.25 (3.79-4.62)	**0.023**
Sodium (mmol/L)	138 (135-140)	138 (135-140)	0.415
Total bilirubin. (mg/dL)	1.28 (0.78-2.34)	1.52 (0.9-2.53)	0.225
Neutrophils (10^9/L)	3.98 (2.48-5.79)	3.51 (2.4-5.23)	0.145
Lymphocytes (10^9/L)	1.27 (0.76-1.83)	1.24 (0.71-1.68)	0.66
Leucocytes (10^9/L)	5.89 (4.22-8.5)	5.4 (4.08-7.57)	0.209
Monocytes (10^9/L)	0.46 (0.34-0.66)	0.44 (0.27-0.59)	0.319
Basophils (10^9/L)	0.02 (0.01-0.04)	0.02 (0.01-0.04)	0.849
Triglycerides (mg/dL)	95 (73-119.5)	89 (68-113.25)	0.199
Glycemia (mg/dL)	105 (91.5-126.5)	100.5 (92.75-123.25)	0.633
Child-Pugh	8 (5.5-9)	7 (6-9)	0.867
MELD score,	11.03 (8.22-16.14)	11.64 (9.25-15.47)	0.389
MELD Na	10.2 (6.48-16.79)	12.17 (7.15-16.77)	0.416
RDW-CV (%)	14.3 (13.45-15.5)	14.3 (13.5-15.8)	0.283
PDW (fL)	16.1 (15.6-16.55)	16.3 (16 - 16.7)	**0.013**
TyG	3.68 (3.57-3.9)	3.69 (3.55-3.83)	0.386
PNI	35.25 (29.0-40.56)	35.34 (30.3-39.77)	0.932

RPR	0.11 (0.07-0.17)	0.14 (0.09-0.22)	**0.013**
NLR	3.43 (1.98-5.81)	2.66 (1.9-5.04)	0.241
dNLR	2.06 (1.42-3.23)	1.71 (1.26-2.83)	0.099
PLR	113.48 (69.75-171.03)	96.47 (58.17-164.24)	0.168
LMR	2.56 (1.65-3.92)	2.9 (1.79-4.39)	0.307
PNR	34.98 (22.54-55.95)	31.41 (21.59-48.94)	0.432
SII	453 (198.35-878.78)	294.49 (161.36-676.36)	**0.028**
AISI	207.8 (71.04-497.81)	119.05 (48.89-325.07)	**0.017**
NLRAR	1.03 (0.52-1.92)	0.78 (0.54-1.52)	0.266
ALBI	-0.94 (-1.64 - -0.22)	-1 (-1.56 - -0.45)	0.913
AST/ALT ratio	1.57 (1.19-2.05)	1.7 (1.2-2.33)	0.312
APRI	1.09 (0.52-1.95)	1.2 (0.67-2.22)	0.116
PALBI	-3.85 (-4 - -3.69)	-3.85 (-3.98 - -3.77)	0.746
FIB-4	4.09 (2.35-6.98)	5.2 (3-8.9)	0.082
ABIC	8.05 (7.48-8.76)	7.71 (7.2-8.44)	**0.035**
NFS	3.12 (2.13-3.86)	3.42 (2.69-3.95)	0.1
King score	26.18 (13.16-60.09)	33.19 (17.02-76.03)	0.2
Lok index	0.84 (0.54-0.98)	0.93 (0.7-0.99)	0.102

Abbreviations: HBV, Hepatitis B virus; HCV, Hepatitis C virus; ALP, alkaline phosphatase; AST, aspartate aminotransferase; ALT, alanine aminotransferase; GGT, γ-glutamyl transferase; PT, prothrombin time; aPTT, activated partial thromboplastin time; INR, international normalized ratio; RDW-CV, red blood cell distribution width-variation coefficient, RPR, RDW-to-platelet ratio; PDW, platelet distribution width; MELD, Model for End-Stage Liver Disease; Lok, cirrhosis probability in hepatitis C ; ABIC, age, serum bilirubin, INR, and serum creatinine; ASP/ALT, aspartate aminotransferase/alanine aminotransferase; NFS, Non-Alcoholic Fatty Liver Disease Fibrosis; FIB-4, fibrosis index, fibrosis-1-index; ASI, aggregate systemic inflammation index; NLR, neutrophil lymphocyte ratio; NLRAR, neutrophil lymphocyte ratio to albumin ratio; PLR, platelet lymphocyte ratio; APRI, AST to platelet ratio index; PALBI, platelet-albumin-bilirubin; dNLR, derived neutrophil-to-lymphocyte ratio; SII, systemic immune-inflammation index; PNI, prognostic nutritional index; TyG, triglyceride glucose index; ALBI, albumin-bilirubin; PNR, platelet-to-neutrophil ratio; LMR, lymphocyte-monocyte ratio.
