# Supplementary material for: Use of Non-Invasive Biomarkers and Clinical Scores to Predict the Complications of Liver Cirrhosis: A Bicentric Experience
Source: Medicina (Kaunas). 2024 Nov 12;60(11):1854. doi: 10.3390/medicina60111854 (PMC11596259; doi:10.3390/medicina60111854)
Supplement: Supplementary file 1 [file medicina-60-01854-s001.zip › medicina-3242154-supplementary-Table S1.pdf]

Table S1. Comparisons of participants characteristics based on the presence of liver cirrhosis' complications.

	**Absence of complications** (***n*=48**)	**Presence of complications** (***n*=187**)	***p*-value**
**Demographic data**			
Age (years), median (IQR)	65 (61-70)	63 (57-70)	0.212
Female gender, n (%)	18 (37)	56 (30)	0.315
**Clinical data, n (%)**			
Alcoholic	29 (60)	108 (58)	0.739
Autoimmune	4 (8)	9 (5)	0.309
Cryptogenic	1 (2)	12 (6)	0.476
Dysmetabolic	3 (6)	18 (10)	0.581
HBV-related	4 (8)	16 (8)	1
HCV-related	10 (20)	32 (17)	0.548
Hemochromatosis	1 (2)	3 (2)	1.00
Mixed	3 (6)	12 (7)	1.00
Ascites	0	88 (47)	**<0.001**
Esophageal and gastric varices	0	10 (5)	0.221
Esophageal varices F1	0	67 (36)	**<0.001**
Esophageal varices F2	0	40 (21)	**<0.001**
Esophageal varices F3	0	12 (6)	0.133
Gastric varices	0	11 (6)	0.126
Hepato-renal syndrome	0	24 (13)	**0.006**
Portal hypertensive gastropathy	0	88 (47)	**<0.001**
Portal vein ectasia	0	36 (19)	**<0.001**
Portal vein thrombosis	0	16 (8)	**0.048**
Presence of varices	0	108 (58)	**<0.001**
Splenomegaly	0	105 (56)	**<0.001**
Spontaneous bacterial peritonitis	0	1 (0.5)	1.00

Laboratory parameters and scores, median (IQR)			
Albumin (g/dL)	3.6 (2.98-4.03)	3.5 (2.9-3.9)	0.548
ALP (UI/L)	89 (69.5-116.5)	109 (78-146)	**0.029**
AST (UI/L)	45.5 (30.25-62.75)	42 (27-73)	0.972
ALT (UI/L)	27.5 (21-41)	26 (18-42.5)	0.434
GGT (UI/L)	87.5 (42.5-158.75)	72 (37-161.5)	0.591
Platelets (10^3/μL)	150 (92-214)	116 (78.5-175)	0.1
PT (s)	14 (11.85-18.05)	14 (12.2-16.9)	0.559
aPTT (s)	31.5 (29.38-35.18)	33.3 (29.8-38)	0.098
INR	1.19 (1.05-1.58)	1.28 (1.1-1.53)	0.185
Fibrinogen (mg/dL)	267.5 (200-330.5)	242 (199-310.5)	0.403
Creatinine (mg/dL)	0.79 (0.64-1)	0.79 (0.66-1.09)	0.68
Potassium (mmol/L)	4 (3.78-4.31)	4.19 (3.74-4.54)	0.195
Sodium (mmol/L)	139 (137-141)	138 (135-140)	**0.015**
Total bilirubin. (mg/dL)	1.12 (0.74-2.4)	1.32 (0.89-2.36)	0.501
Neutrophils (10^9/L)	4.14 (2.56-5.73)	3.56 (2.41-5.49)	0.28
Lymphocytes (10^9/L)	1.42 (0.98-1.89)	1.22 (0.71-1.69)	**0.039**
Leucocytes (10^9/L)	6.17 (4.99-8.32)	5.45 (3.85-7.76)	0.06
Monocytes (10^9/L)	0.13 (0.07-0.2)	0.11 (0.05-0.17)	0.256
Basophils (10^9/L)	0.03 (0.02-0.04)	0.02 (0.01-0.04)	0.209
Triglycerides (mg/dL)	93.5 (81.5-109.5)	91 (68.5-118.5)	0.56
Glycemia (mg/dL)	99 (91-131)	104 (92.5-124)	0.835
Child-Pugh	6.5 (5-8)	8 (6-9.5)	**<0.001**
MELD score,	10.82 (7.64-17.52)	11.33 (8.8-15.37)	0.663
MELD Na	9.09 (6.03-16.5)	11.84 (7.18-16.94)	0.195
RDW-CV (%)	13.95 (13-14.62)	14.5 (13.6-15.85)	**0.002**
PDW (fL)	16.2 (15.8-16.42)	16.2 (15.7-16.65)	0.438
TyG	3.71 (3.54-3.89)	3.68 (3.56-3.84)	0.777
PNI	36.94 (30.65-41.15)	35.25 (29.46-39.94)	0.438
RPR	0.09 (0.06-0.15)	0.13 (0.08-0.19)	**0.019**

NLR	2.96 (1.69-4.4)	3.1 (2-5.67)	0.368
dNLR	1.92 (1.25-2.84)	1.97 (1.39-3.04)	0.687
PLR	95.16 (66.43-148.44)	102.16 (65.04-170.08)	0.511
LMR	2.76 (2.09-3.72)	2.79 (1.69-4.34)	0.951
PNR	29.66 (22.15-54.62)	32.81 (22.04-50.48)	0.938
SII	409.9 (205.97-825.98)	385.78 (171.16-790.32)	0.742
AISI	197.6 (84.02-458.5)	141.76 (54.62-448.32)	0.256
NLRAR	0.84 (0.45-1.52)	0.97 (0.54-1.73)	0.307
ALBI	-1.16 (-1.78-0.17)	-0.99 (-1.56 - -0.33)	0.794
AST/ALT ratio	1.51 (1.14-1.87)	1.67 (1.21-2.33)	0.099
APRI	1.11 (0.53-1.93)	1.19 (0.58-2.1)	0.388
PALBI	-3.87 (-3.99 - -3.71)	-3.85 (-3.99 - -3.72)	0.618
FIB-4	4.04 (2.32-6.45)	5.14 (2.55-8.13)	0.185
ABIC	8.18 (7.59-8.82)	7.84 (7.35-8.49)	0.09
NFS	2.91 (2.11-3.8)	3.34 (2.56-3.87)	0.162
King score	23.85 (12.96-60.91)	32.4 (15.22-65.03)	0.467
Lok index	0.77 (0.52-0.95)	0.92 (0.67-0.99)	**0.032**

Abbreviations: HBV, Hepatitis B virus; HCV, Hepatitis C virus; ALP, alkaline phosphatase; AST, aspartate aminotransferase; ALT, alanine aminotransferase; GGT, γ-glutamyl transferase; PT, prothrombin time; aPTT, activated partial thromboplastin time; INR, international normalized ratio; RDW-CV, red blood cell distribution width-variation coefficient, RPR, RDW-to-platelet ratio; PDW, platelet distribution width; MELD, Model for End-Stage Liver Disease; Lok, cirrhosis probability in hepatitis C ; ABIC, age, serum bilirubin, INR, and serum creatinine; ASP/ALT, aspartate aminotransferase/alanine aminotransferase; NFS, Non-Alcoholic Fatty Liver Disease Fibrosis; FIB-4, fibrosis index, fibrosis-1-index; ASI, aggregate systemic inflammation index; NLR, neutrophil lymphocyte ratio; NLRAR, neutrophil lymphocyte ratio to albumin ratio; PLR, platelet lymphocyte ratio; APRI, AST to platelet ratio index; PALBI, platelet-albumin-bilirubin; dNLR, derived neutrophil-to-lymphocyte ratio; SII, systemic immune-inflammation index; PNI, prognostic nutritional index; TyG, triglyceride glucose index; ALBI, albumin-bilirubin; PNR, platelet-to-neutrophil ratio; LMR, lymphocyte-monocyte ratio.
